# First report on prevalence of SARS-CoV-2 infection among health-care workers in Nicaragua

**DOI:** 10.1371/journal.pone.0246084

**Published:** 2021-01-27

**Authors:** Jorge A. Huete-Pérez, Cristiana Cabezas-Robelo, Lucía Páiz-Medina, Carlos A. Hernández-Álvarez, Carlos Quant-Durán, James H. McKerrow

**Affiliations:** 1 Molecular Biology Center, University of Central America, UCA, Managua, Nicaragua; 2 Nicaraguan Multidisciplinary Scientific Committee on COVID-19, Managua, Nicaragua; 3 Metabolic and Infectious Diseases, Vivian Pellas Hospital, Masaya, Nicaragua; 4 Center for Discovery and Innovation in Parasitic Diseases, Skaggs School of Pharmacy and Pharmaceutical Sciences, University of California San Diego, La Jolla, CA, United States of America; UCLA Fielding School of Public Health, UNITED STATES

## Abstract

The Nicaraguan COVID-19 situation is exceptional for Central America. The government restricts testing and testing supplies, and the true extent of the coronavirus crisis remains unknown. Dozens of deaths have been reported among health-care workers. However, statistics on the crisis’ effect on health-care workers and their risk of being infected with SARS-CoV-2 are lacking. We aimed to estimate the prevalence of SARS-CoV-2 infection in health-care workers and to examine correlations with risk factors such as age, sex and comorbidities. Study participants (N = 402, median age 38.48 years) included physicians, nurses and medical assistants, from public and private hospitals, independent of symptom presentation. SARS-CoV-2 was detected on saliva samples using the loop-mediated isothermal amplification assay. A questionnaire was employed to determine subjects’ COVID-19-associated symptoms and their vulnerability to complications from risk factors such as age, sex, professional role and comorbidities. The study was performed five weeks into the exponential growth period in Nicaragua. We discovered that 30.35% of health-care workers participating in our study had been infected with SARS-CoV-2. A large percentage (54.92%) of those who tested positive were asymptomatic and were still treating patients. Nearly 50% of health-care workers who tested positive were under 40, an astonishing 30.33% reported having at least one comorbidity. In our study, sex and age are important risk factors for the probability of testing positive for SARS-CoV-2 with significance being greatest among those between 30 and 40 years of age. In general, being male resulted in higher risk. Our data are the first non-governmental data obtained in Nicaragua. They shed light on several important aspects of COVID-19 in an underdeveloped nation whose government has implemented a herd-immunity strategy, while lacking an adequate healthcare system and sufficient PPE for health-care workers. These data are important for creating policies for containing the spread of SARS-CoV-2.

## Introduction

The novel coronavirus disease 2019 (COVID-19) pandemic has overtaxed public health systems worldwide, and health-care workers (HCW) are at elevated risk of contracting the disease due to frequent and proximate contact to patients with COVID-19 [1, 2].

In Nicaragua, dozens of deaths have been reported among HCW and numbers are quickly rising [3, 4]. Due to government restrictions, the true extent of the coronavirus crisis in Nicaragua is unknown, and statistics on the impact of the crisis on HCW and their risk of being infected with Severe Acute Respiratory Syndrome Coronavirus–2 (SARS-CoV-2), are lacking [5]. In the absence of effective containment measures [6] the virus has spread to a large portion of the population due to the highly infectious nature of SARS-CoV-2 and transmission of the virus by asymptomatic and presymptomatic individuals [7]. The Pan American Health Organization has decried having to rely on data from independent sources for insight into the country´s coronavirus pandemic and has called for an independent evaluation [8].

Enabling rapid identification and isolation of infected HCW is crucial for maintaining the health and safety of front line workers, and protecting patients and the broader community as a whole. Yet despite growing awareness of a lack of data, testing is severely limited by the government and restricted to only one government-operated facility.

In this study, we aimed to estimate the prevalence of SARS-CoV-2 infection in a population of HCW, 14–18 weeks following Nicaragua’s first COVID-19 case and to examine correlations with risk factors such as age, gender and comorbidities.

## Materials and methods

### Study population and settings

A total of 402 subjects (256 women, 146 men) were enrolled in the study with a median age of 38.48 years (20–78 years), independent of symptom presentation. We prioritized health-care workers (HCW) working directly with patients.

A questionnaire was employed to determine clinical symptoms most commonly associated with COVID-19 and their vulnerability to severe illness and complications from infection in relation to risk factors such as age, sex, professional role and chronic medical conditions. The questionnaire also included use of personal protective equipment (PPE) and presence of a family member with COVID-19 symptoms at home. It was developed by the research team after thorough literature review and in consultation with a group of physicians not involved in the study. A pre-testing of the questionnaire was carried out on the first 15 enrolled participants of different age, gender and professional roles. One item was eliminated while 4 items were modified according to the participants’ recommendations, producing the 34-item final questionnaire. On the day of the specimen collection during the study period, an investigator in charge of recruiting volunteers would invite hospital staff at the selected hospitals. Interested volunteers received an explanation on the purpose of the study, how to fill out the questionnaire and were asked to sign to indicate their informed-consent. The study’s HCW included physicians (208), nurses (154) and medical assistants (40), from public and private hospitals (44 and 358 subjects respectively). The sample is representative of the city of Managua, the most populous city of Nicaragua, where the study took place and, although it included HCW from anywhere in the country, it may not be representative of the situation in the whole country. Our study was conducted according to the principles expressed in the Declaration of Helsinki, was reviewed and approved by the Ethics Committee of the University of Central America and written informed consent was obtained from all participants.

### Specimen collection and testing

Between 1 and 4 milliliters of saliva were self-collected by each study participant in flasks containing 2 milliliters of sample buffer [9] (1x Phosphate Buffered Saline, pH 7) for SARS-CoV-2 Loop-mediated isothermal amplification (LAMP) detection. After collection, specimens were sent on ice to the Molecular Biology Center of the University of Central America for the detection of nucleic acids from SARS-CoV-2. Assays were performed on saliva samples without an RNA purification step within 1–2 hours following collection. All LAMP reactions were performed following New England Biolab’s (NEB) published protocol using WarmStart Colorimetric LAMP 2X Master Mix (NEB, M1800L) [10, 11]. We used 20 μL reactions consisting of 10 μL of 2X master mix, 2 μL of 10X primer mix targeting the viral genes N and E (2 μM F3 and B3, 16 μM Forward Inner Primer (FIP) and Backward Inner Primer (BIP), and 4 μM Loop Forward (LF) and Loop Backward (LB) primers), 5 μL nuclease-free water, and 3 μL samples (NEB E2019 COVID LAMP kit). LAMP reactions were incubated at 65°C using an Applied Biosystems 2720 Thermal Cycler for 45 minutes. Photographs were taken with cell phone cameras of the testing tubes laid on white sheets of paper for visual color-based detection of amplification reactions, with pink indicating a negative result and yellow indicating positive detection of SARS-CoV-2 result. For quality control and to validate the LAMP methodology in our laboratory, we performed 12 additional saliva tests on individuals who had been diagnosed as SARS-CoV-2 positive by quantitative reverse transcription PCR (qRT-PCR) using nasopharyngeal swabs at the central government laboratory. Our saliva LAMP results showed total concordance with those obtained by the government laboratory by qRT-PCR. These 12 volunteer patients (5 males and 7 females) were identified among university staff. All of them presented mild or moderate cases of COVID-19 disease, except for one critically ill individual who eventually died.

## Statistical analysis

To analyze our data, we prepared a Microsoft® Excel® document with the results of SARS-CoV-2 detection using the LAMP assay, along with data obtained from the questionnaire. Prior to statistical analyses, all data were transferred to a spreadsheet without linkage to the original database, eliminating all personal identification data. We analyzed the associations of SARS-CoV-2 detection results (positive or negative) with age, sex, professional role, comorbidities, and having a family member with COVID-related symptoms. Tables of the most representative associations were prepared with frequency, percentage, chi-squared values (X^2^), probability (*P*), and regressions, provided as odds ratios (OR) and corresponding 95% confidence intervals (CI). Variables with *P* values of ≤0.05 were considered statistically significant. SPSS (Statistical Package for the Social Sciences) statistical software version 22.0 was used for the association analysis.

## Results

SARS-CoV-2 was detected in 122 (30.35%) of the 402 subjects during the study period (June 22 to July 22, 2020). Results were conveyed to subjects within 24 hours via phone calls and advice on self-isolation was provided. Of 256 female subjects, 75 (29.30%) tested positive. Of 146 male subjects, 47 (32.19%) tested positive (Table 1).

**Table 1:** Demographic data of health-care workers.

Category	Physicians	Nurses	Medical assistants^a^	Total
Number	208	154	40	402
Percentage	51.74%	38.31%	9.95%	100.00%
Median age (years)	42.55	33.86	34.63	38.48
Sex (male/female)	105/103	29/125	12/28	146/256
Number of positive results	67 (32.21%)	42 (27.27%)	13 (32.50%)	122 (30.35%)

^a^Medical assistants: radiology technicians, bioanalysts, and laboratory technicians in direct contact with COVID-19 patients.

The mean ages of those testing positive (39.70 years [SD 12.73]) or negative (37.91 years [SD 12.78]) were close (t test p = 0.196) but slightly higher for those testing positive. The highest infection rate was in physicians, 32.21% and medical assistants, 32.50%. Nurses had a lower infection rate, 27.27%. The prevalence of positive results for HCW from private hospitals was 29.33% (105) and 38.64% (17) from public hospitals. HCW aged 20–29 formed the largest group testing positive, followed by those aged 40–49 (Table 2).

**Table 2:** Number of SARS-CoV-2 positive subjects by age group and sex.

Sex	Age group (years)						Total
	20–29	30–39	40–49	50–59	60–69	70+	
Male	13 (27.66%)	9 (19.15%)	11 (23.40%)	8 (17.02)	5 (10.64%)	1 (2.13%)	47
Female	23 (30.67%)	17 (22.67%)	19 (25.33%)	12 (16.00%)	4 (5.33%)	0 (0.00%)	75
Total	36 (29.51%)	26 (21.31%)	30 (24.59%)	20 (16.39%)	9 (7.38%)	1 (0.82%)	122

The most common symptoms reported by those testing positive were headache (22.95%), body aches/discomfort (15.57%), cough (13.43%), sore throat (13.93%), fever (12.30%), loss of smell and taste (9.84%) and asthenia (8.20%). Diarrhea was reported by 8.20%. SARS-CoV-2 positive HCW self-reported several symptoms more frequently than those with negative assays: headache (22.95% vs. 12.50%), and body aches and discomfort (15.57% vs. 7.14%) (all *P*<0.05). Sixty-seven SARS-CoV-2-positive HCW (54.92%) were asymptomatic (Table 3 and S1 Table). Most asymptomatic subjects (58.3%) were younger than 50 years old.

**Table 3:** Symptoms among health-care workers by SARS-CoV-2 test results^a^.

	Overall (N = 402)	Positive (N = 122)	Negative (N = 280)	P value
Males	146 (36.32%)	47 (38.52%)	99 (35.36%)	0.548
Females	256 (63.68%)	75 (61.48%)	181 (64.64%)	0.548
No symptoms	262 (65.17%)	67 (54.92%)	195 (69.64%)	** 0.006**
At least one COVID-19 symptom	140 (34.83%)	55 (45.08%)	85 (30.36%)	** 0.006**
Headache	63 (15.67%)	28 (22.95%)	35 (12.50%)	** 0.016**
Body aches and discomfort	39 (9.70%)	19 (15.57%)	20 (7.14%)	** 0.022**
Cough	43 (10.70%)	17 (13.93%)	26 (9.29%)	0.198
Sore throat	46 (11.44%)	17 (13.93%)	29 (10.36%)	0.327
Fever	35 (8.71%)	15 (12.30%)	20 (7.14%)	0.127
Loss of smell and gustatory dysfunction	30 (7.46%)	12 (9.84%)	18 (6.43%)	0.270
Asthenia	20 (4.98%)	10 (8.20%)	10 (3.57%)	0.092
Diarrhea	30 (7.46%)	10 (8.20%)	20 (7.14%)	0.720

^a^All tests were two-sided and a *P*-value ≤ 0.05 was considered statistically significant.

The most common comorbidities reported among all 402 subjects were hypertension (72; 17.91%), obesity (22; 5.47%), and diabetes (19; 4.73%). Other reported illnesses included cancer, cardiovascular diseases, autoimmune diseases, arthritis, gastritis and asthma (Table 4). Among subjects who tested positive for SARS-CoV-2, the most common comorbidities were hypertension (22; 18.03%), obesity (9; 7.38%), and diabetes (9; 7.38%).

**Table 4:** Preexisting medical conditions in health-care workers by SARS-CoV-2 test results.

Preexisting medical conditions	Overall (N = 402)	Positive (N = 122)	Negative (N = 280)
At least one preexisting medical condition	102 (25.37%)	37 (30.33%)	65 (23.21%)
Hypertension	72 (17.91%)	22 (18.03%)	50 (17.86%)
Obesity	22 (5.47%)	9 (7.38%)	13 (4.64%)
Diabetes	19 (4.73%)	9 (7.38%)	10 (3.57%)
Cancer	8 (1.99%)	4 (3.28%)	4 (1.43%)
Cardiovascular diseases	4 (1.00%)	2 (1.64%)	2 (0.71%)
Autoimmune diseases	4 (1.00%)	2 (1.64%)	2 (0.71%)

Three hundred and seventy-six HCW reported using PPE during the study period; 94.97% at private hospitals and 81.82% at public hospitals. Only 24 HCW (5.97%) reported not using PPE at their hospital. The questionnaire did not enquire about specific type of PPE used, receipt of PPE training or the practice of other personal protective measures (S2 Table). Of 402 subjects, 93 (23.13%) reported a family member with COVID-19 symptoms at home, while of 122 subjects testing positive, 39 (31.96%) reported a family member with COVID-19 symptoms at home.

In this study, we determined the associations of SARS-CoV-2 infection with age, sex, professional role, comorbidities, and having a family member with COVID-related symptoms. HCW age was associated with the probability of testing positive for SARS-CoV-2. When comparing HCW in the 40 years old or older group with the under 40 age group, the prevalence of SARS-CoV-2 positivity was 39.2% and 27.0% respectively (*P* = 0.009) (Table 5 and S3 Table). HCW ≥ 40 years old had a 70% greater probability (OR 1.7) of testing positive compared to HCW < 40 years of age. The difference in prevalence of positivity between the two age groups in men was minor (34.2% and 33.3%; *P* = 0.527); whereas in women, the prevalence of positivity for those ≥ 40 was 43.2% and for those < 40 was 24.4% (*P* = 0.002; OR 2.4). Similarly, professional role at the hospital was associated with SARS-CoV-2 infection. Physicians ≥ 40 were more likely to test positive (80% greater probability) than their younger peers (OR 1.8; 95% CI, 1.0–3.3). No significant relationship between professional role and SARS-CoV-2 infection was found for nurses and medical assistants.

**Table 5:** Association of SARS-CoV-2 infection with risk factors among health-care workers in Nicaragua.

Category	Number	Positivity rates, n/N^a^ - %		X^2^	P-value	OR	95%CI
HCW ≥ 40 years Vs. HCW < 40 years		≥ 40 years	<40 years				
Total^b^	384	60/154–39.2%	62/230–27.0%	6.132	**0.009**	1.7	1.1–2.7
Males	139	25/73–34.2%	22/66–33.3%	0.013	0.527	1.0	0.5–2.1
Females	245	35/81–43.2%	40/164–24.4%	9.041	**0.002**	2.4	1.3–4.1
Physicians	201	44/113–38.9%	23/88–26.1%	3.648	**0.039**	1.8	1.0–3.3
Nurses and medical assistants	183	16/41–39.0%	39/142–27.5%	2.022	0.178	1.7	0.8–3.5
Comorbidities	96	30/75–40.0%	8/21–38.1%	0.025	1.000	1.1	0.4–2.9
No comorbidities	288	30/79–38.0%	54/209–25.8%	4.008	**0.032**	1.8	1.1–2.7
Asymptomatics Vs.at least one COVID-19 symptom		Asymptomatics	At least one symptom				
Total	402	67/263–25.5%	55/139–39.6%	8.545	**0.004**	0.5	0.3–0.8
Males	146	23/94–24.5%	24/52–46.2%	7.213	**0.010**	0.4	0.2–0.8
Females	256	44/169–26.0%	31/87–35.6%	2.554	0.114	0.6	0.4–1.1
HCW with comorbidities Vs. HCW without comorbidities		HCW with comorbidities	HCW without comorbidities				
Total	384	38/96–39.6%	84/288–29.2%	3.604	**0.039**	1.6	1.0–2.6
HCW ≥50 years	82	16/47–34.0%	14/35–40.0%	0.307	0.646	0.8	0.3–1.9
HCW <50 years	302	22/49–44.9%	70/253–27.7%	5.753	**0.026**	2.1	1.1–4.0
HCW with obesity Vs. HCW without obesity		HCW with obesity	HCW without obesity				
Total	402	9/22–40.9%	113/380–29.7%	1.228	0.339	1.6	0.7–3.9
Physicians	208	7/12–58.3%	60/196–30.6%	3.979	**0.050**	3.2	1.0–10.4
Nurses and medical assistants	194	2/10–20.0%	53/184–28.8%	0.362	0.728	0.6	0.1–3.0
HCW with symptomatic family members Vs. HCW without symptomatic family members		HCW with symptomatic family members	HCW without symptomatic family members				
Total	402	39/94–41.5%	83/308–26.9%	7.204	**0.010**	1.9	1.2–3.1
Males	146	12/30–40.0%	35/116–30.2%	1.055	0.381	1.5	0.7–3.5
Females	256	27/64–42.2%	48/192–25.0%	6.846	**0.011**	2.2	1.2–4.0
Physicians	208	24/53–45.3%	43/155–27.7%	5.565	**0.026**	2.2	1.1–4.1
Nurses and medical assistants	194	15/41–36.6%	40/153–26.1%	1.736	0.241	1.6	0.8–3.4
HCW ≥30 years	267	26/60–43.3%	60/207–29.0%	4.386	**0.042**	1.9	1.0–3.4
HCW <30 years	117	13/29–44.8%	23/88–26.1%	3.577	**0.050**	2.3	1.0–5.5
Comorbidities	103	12/20–60.0%	26/83–31.3%	5.692	**0.022**	3.0	1.1–8.4
No comorbidities	299	27/74–36.5%	57/225–25.3%	3.429	**0.046**	1.7	1.0–3.0
Hypertension	72	7/12–58.3%	15/60–25.0%	5.236	**0.037**	3.3	1.2–9.0
Without Hypertension	330	32/82–39.0%	68/248–27.4%	3.93	**0.034**	1.7	1.0–2.9

^a^n: number of positive results; N: total number.

^b^ Three hundred and eighty-four HCW out of 402 declared their age.

The presence of comorbidities among HCW of all ages is linked to high prevalence of SARS-CoV-2 positivity (40.0% for ≥ 40 and 38.1% for < 40); however, age did not affect probability (*P* = 1.000). Nevertheless, for those with no reported comorbidities, age was related to risk (*P* = 0.032), for HCW ≥ 40 without comorbidities, prevalence was 38.0% and for HCW <40, without comorbidities, prevalence was only 25.8%. Thus, older HCW have an 80% greater probability of testing positive for SARS-CoV-2 compared to younger HCW (OR 1.8; 95% CI, 1.1–2.7).

Asymptomatic HCW were less likely to test SARS-CoV-2 positive than HCW with one or more COVID-19-related symptoms (25.5% and 39.6% respectively). This effect was more evident in male HCW (24.5% in asymptomatic and 46.2% in symptomatic). The presence of one or more comorbidities increased the probability of testing positive for SARS-CoV-2 by 60%, compared to those who reported an absence of comorbidities (*P* = 0.039; OR 1.6; 95% CI, 1.0–2.6). For those HWC ≥ 50 years old who reported comorbidities, the probability of testing positive for SARS-CoV-2 double (*P* = 0.026, OR 2.1; 95% IC, 1.1–4.0). This association was not detected in the group < 50. Although in our study, obesity was one of the most frequent comorbidities (22/402, 5.47%), no direct association with the probability of testing positive for SARS-CoV-2 was detected when combining all ages, sex and professions (*P* = 0.339). However, the probability of a positive test result tripled for physicians with obesity compared to their non-obese peers (*P* = 0.050; OR 3.2; 95% CI, 1.0–10.4). This association was not detected in nurses and medical assistants (p = 0.728).

A strong association (p = 0.010) was found between those HCW who have family members with COVID-19-related symptoms at home and the probability of testing positive for SARS-CoV-2 (OR 1.9; 95% CI, 1.2–3.1) compared with those who have no one at home with related symptoms. This association was even greater (triple) for those HCW who reported a comorbidity (*P* = 0.022) and a symptomatic relative at home, compared to those who had no symptomatic relatives at home. For those with hypertension, and a symptomatic relative at home, the probability of testing positive triple (*P* = 0.037; OR 3.3; 95% CI, 1.2–9.0).

## Discussion

To our knowledge, this is the first and only study to date presenting data obtained from testing of SARS-CoV-2 infection in the Nicaraguan population. It provides important, previously unknown insights into the COVID-19 epidemic in Nicaragua.

We discovered that between June 22 and July 22, 2020, 30.35% of HCW participating in our study were actively infected with SARS-CoV-2 RNA. These findings reveal that the overall prevalence is relatively high. Studies in various European countries observe lower infection rates [12, 13] among symptomatic healthcare workers, attributed to observance of rigorous hygiene standards, [2] but seroprevalence of SARS-CoV-2 in different hospital settings can range from 0.8% to 31.2% [14, 15].

**Fig 1:**
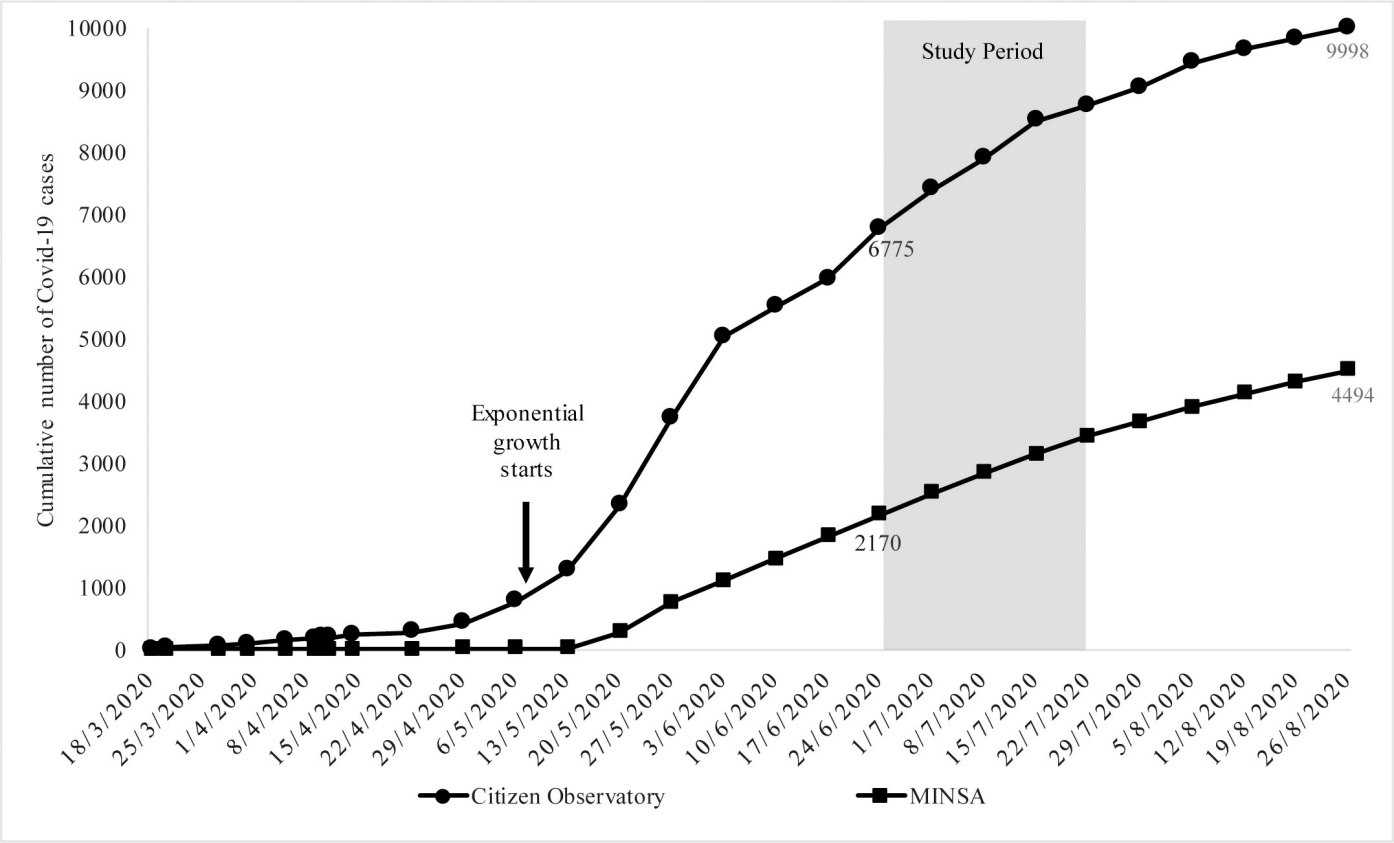
Cumulative number of COVID-19 cases in Nicaragua. The arrow indicates the beginning of the exponential growth around May 6. The shaded area shows the period of the study (June 24 to July 22, 2020), about five weeks into the exponential growth period. Note the difference between the data provided by the independent Citizen Observatory (top curve) [https://observatorioni.org/estadisticas-covid-19-nicaragua/] and the official data registered weekly (instead of daily) by the Nicaraguan Ministry of Health (MINSA) and reported by the Johns Hopkins University Center for Systems Science and Engineering (CSSE) [https://coronavirus.jhu.edu/region/nicaragua]. SARS-CoV-2 = severe acute respiratory syndrome coronavirus 2. COVID-19 = coronavirus disease 2019.

The first case of COVID-19 in Nicaragua was reported on March 18, 2020. Our study was performed about five weeks into the exponential growth period (Fig 1), during widespread community transmission in many parts of Nicaragua, overwhelming hospitals and putting HCW at risk of infection [16]. On August 26, 161 days after that first diagnosed case, the government admitted a total of only 4,494 cases, with only 137 COVID-19-related deaths. At the same time, the Citizen Observatory reported a total of 9,998 positive coronavirus cases and 2,680 COVID-19-related deaths, which for a 6.4 million population gives a rate of 419 COVID-19 deaths per million people, among the highest in Latin America.

In our study, sex and age are important risk factors for the probability of testing positive for SARS-CoV-2- with significance being greatest among those between 30 and 40 years of age. Men were at slightly higher risk than women (32.19% for men and 29.30% for women). Subjects in the 20–29 age group were the most affected by SARS-CoV-2 infection (36; 29.51%). Other studies have shown similar prevalence of infection among young HCW. In South Korea 26.9% of cases occurred among HCW aged 20–29 years [17, 18]. Risk increases when comorbidities are present, which poses important implications for occupational infections of HCW in hospital settings. Risk of testing positive for SARS-CoV-2 also increases when there are relatives at home with COVID-19-related symptoms, a situation reported by many of the health-care workers in this study.

As we anticipated, the prevalence of SARS-CoV-2 infection was higher for HCW in public hospitals (38.64%) than in private hospitals (29.33%). HCW at public hospitals attend the most vulnerable populations with the least resources, and the use of surgical mask, face shield, gloves, gown or any other kind of protection equipment is frequently discouraged by authorities, justified as an attempt to avoid provoking panic among patients [19–21]. Accordingly, patients were not required to wear face masks at hospitals either, thus posing further risks to HCW [19]. Moreover, in the early stage of the pandemic, health professionals complained that they were not provided with PPE and that they had to supply their own PPE [19–21]. The Pan American Health Organization called on the Nicaraguan authorities to provide appropriate PPE for HCW [22]. In response to a lack of official measures to contain the spread of the virus, such as social distancing, mask wearing or PPE usage, 34 medical associations called for a voluntary national quarantine [23]. However, because of the environment in which the study was carried out and to keep the questionnaire as brief as possible for rapid turn-around, the kind of PPE used and the timeline for its availability could not be determined. For epidemiological reasons, it is critically important to look at how the different neighboring countries addressed the coronavirus pandemic, particularly their guidelines on PPE usage, and to explore the prevalence of COVID-19 cases. This is particularly useful considering that Nicaragua, in contrast to the rest of Central America, did not follow the Pan American Health Organization's recommendations [24], however we are not aware of any scientific studies on the prevalence of SARS-CoV-2 among HCW in Central America. Regarding other Latin American countries such as Brazil, Colombia and Ecuador, an international survey reports a lack of access to different PPE equipment and diagnostic methods for both professionals and patients, and that 70% of professionals stated not having the necessary resources to care for patients with COVID-19 [25]. The scarcity of PPE and lack of training has been determined as a risk factor for the high number of SARS-CoV-2 infections among HCW [26, 27].

The most significant initial symptoms reported by those testing positive were headache (22.95%), body aches/discomfort (15.57%), cough (13.93%), sore throat (13.93%) and fever (12.30%), which are considered the most common symptoms associated with COVID-19 [18, 28]. Among all those infected with SARS-CoV-2, 54.92% were asymptomatic. Some 58.33% of those under 50 were asymptomatic, and 48.15% of those over 50 were asymptomatic. In contrast, in a cohort study among HCW in Italy, almost a third of COVID-19 cases were asymptomatic [29]. In a recent study in 13 U.S. academic medical centers, approximately one third of HCW who tested positive for SARS-CoV-2 antibodies did not report any COVID-19-associated symptoms [14]. Asymptomatic individuals may account for up to 40% to 45% of SARS-CoV-2 infections [30–32]. Unaware of being infected, asymptomatic HCW continue their normal work routine. Identifying asymptomatic COVID-19-positive individuals provides crucial epidemiological information for preventing and controlling the spread of the SARS-CoV-2, especially healthcare-associated outbreaks. Early detection within hospitals should be prioritized through effective rapid and proactive testing [33], followed by isolation.

Nearly 50% of HCW in our study who tested positive for SARS-CoV-2 were under 40 (median age 38.48 years), and an astonishing 30.33% reported having at least one comorbidity, with hypertension, obesity and diabetes being the most common. This finding is critical for evaluating the true risk of serious adverse outcomes in Nicaraguan HCW diagnosed with COVID-19, as the risk of suffering from serious complications due to COVID-19 when chronic medical conditions are also present is shared by individuals of all ages [34–36]. Early detection among HCW is crucial not only for protecting patients but to maximize the available health-care workforce [37] and to prevent nosocomial SARS-CoV-2 infections [38] in settings with confirmed COVID-19.

Our study found 30.35% of HCW to be infected with SARS-CoV-2. Due to a number of factors, it was not possible to determine the percentage of all Nicaraguan HCW who have been infected with the virus. Nicaraguan HCW should undergo frequent testing for SARS-CoV-2 to assess the effect of the epidemic on healthcare personnel, slow the spread of infection within health centers and lessen the burden on the public health system.

The Nicaraguan COVID-19 situation is exceptional for Central America. Keeping tight control over all data, the government prohibits independent COVID-19 testing. Data from the limited government-controlled testing, performed at one government laboratory, are scant, reported inaccurately and considered unreliable by international standards.

Our study provides the first non-governmental data on SARS-CoV-2 infection rates. The LAMP assay used here as a detection technique may allow for faster and cheaper testing. Other advantages that make it attractive for point-of-care or epidemiological convenience include its reported sensitivity of 97.5% and a specificity of 99.7% compared to qRT-PCR [39, 40], within a growing field of application variations [41–47]. Because it detects the SARS-CoV-2 virus directly from saliva and requires only a heating instrument instead of a thermocycler apparatus, it is more suitable, convenient and applicable for early case detection and isolation in most Nicaraguan hospitals. Saliva specimens may have at least comparable sensitivity and are less invasive than nasopharyngeal swab specimens in detecting SARS-CoV-2 in hospital settings [48–51] and can be used successfully even in asymptomatic persons [50, 52, 53]. Given the scarcity and the inefficiency of government testing, the methodology used in our study could be applied in public health surveillance by supplementing or replacing established qRT-PCR diagnostic testing, limited by capacity and cost. Given the importance of understanding the evolving epidemiology and transmission dynamics of the outbreak [54], these data and methodology procedures could also be of great benefit to other developing countries with limited resources and the urgent need for rapid testing as a way to guide intervention policy to contain the spread of the virus.

## Conclusions

Our dataset is highly informative, describing the symptoms, comorbidities and infection rate among public and private HCW during a one-month period, of whom 30.35% were infected with SARS-CoV-2, while continuing to see patients and lacking appropriate and sufficient PPE. Further research will determine if this infection rate could be used as a reference for the currently practicing Nicaraguan HCW population as a whole. Our results shed light on several important and timely aspects of SARS-CoV-2 in a poor, underdeveloped nation whose government has implemented a herd immunity approach to confronting the COVID-19 pandemic; and where the lack of diagnostic tests and truthful reporting of data are two of the greatest limitations. Our data are important for those seeking to understand the rapid and expansive spread of the virus within vulnerable communities lacking effective policies for protection from and containment of the SARS-CoV-2 pandemic. As COVID-19 surges in Latin America, lessons learned in Nicaragua may be applicable in other low-income countries with similar vulnerabilities.

## Supporting information

S1 Table Prevalence of SARS-CoV-2 among health-care workers in Nicaragua, symptoms and comorbidities database. (XLSX)

S2 Table Questionnaire used in this study. (PDF)

S3 Table Association analysis on SARS-CoV-2 infection and risk factors. (XLSX)
