# Supplementary material for: First report on prevalence of SARS-CoV-2 infection among health-care workers in Nicaragua
Source: PLoS One. 2021 Jan 27;16(1):e0246084. doi: 10.1371/journal.pone.0246084 (PMC7840011; doi:10.1371/journal.pone.0246084)
Supplement: S2 Table — (PDF) [file pone.0246084.s002.pdf]

Prueba de COVID-19 por técnica molecular LAMP.
Estudio internacional de incidencia de SARS-CoV-2 en el personal de salud.
Ficha y autorización

Ficha número: ___________

I. Datos generales

Nombre:

Edad:

Sexo: ☐ **M** ☐ **F**

Fecha toma de muestra: **Hora:**

Profesión u oficio: Médico ( ), Personal de Enfermería ( ), especialista en: ________________________________

Unidad de salud:

Número celular para aviso de resultado:

II. Síntomas clínicos (marcar según corresponda)

☐ Sin síntomas
☐ Fiebre
☐ Tos seca
☐ Cansancio
☐ Dificultad para respirar
☐ Dolor o presión en el pecho
☐ Dolor de garganta
☐ Dolor de cabeza
☐ Conjuntivitis
☐ Diarrea
☐ Pérdida del sentido del olfato o del gusto
☐ Incapacidad para hablar o moverse
☐ Molestias y dolores
☐ Fecha aparición de síntomas: ______________________

Otros: ____

III. Antecedentes clínicos

☐ **Hipertensión** ☐ **Diabetes** ☐ **Enfermedad coronaria**
☐ **Obesidad** ☐ **Cáncer** ☐ **Enfermedades autoinmunes** **Otros:** _____________

En su trabajo, ¿cuenta con equipos de protección personal? Sí ☐ No ☐

¿Hay algún enfermo con síntomas de COVID-19 en su núcleo familiar? Sí ☐ No ☐

Observaciones:

____

Firma: ________________________________

Autorizo de forma voluntaria que se analice mi muestra en el estudio de detección molecular del virus.

COVID-19 test by LAMP molecular technique
International study on the incidence of SARS-CoV-2 in HCW
File and authorization

Id number: ___________

I. General information

Name:

Age:

Sex: ☐ **M** ☐ **F**

Sample collection date: **Time:**

Profession: Physician ( ), Nurse ( ), specialist in: ____

Hospital

Cell phone number to notify the result:

II. Clinical symptoms (mark accordingly)

☐ No symptoms	☐ Headache
☐ Fever	☐ Conjunctivitis
☐ Cough	☐ Diarrhea
☐ Asthenia	☐ Loss of smell and gustatory dysfunction
☐ Difficulty breathing or shortness of breath	☐ Loss of speech or movement
☐ Chest pain or pressure	☐ Body aches and discomfort
☐ Chest pain or pressure	☐ Date of onset of symptoms: ____________________

Others: ____

III. Clinical history

☐ **Hypertension**	☐ **Diabetes**	☐ **Cardiovascular diseases**
☐ **Obesity**	☐ **Cancer**	☐ **Autoimmune diseases** **Others:** ____________

At your work place, do you have PPE available? Yes ☐ No ☐

Do you currently have a family member with COVID-19 symptoms at home? Yes ☐ No ☐

Observations: ____

Signature: ______________________________

I voluntarily authorize my sample to be analyzed in the molecular virus detection study
